# Serotonin versus catecholamine deficiency: behavioral and neural effects of experimental depletion in remitted depression

**DOI:** 10.1038/tp.2015.25

**Published:** 2015-03-17

**Authors:** P Homan, A Neumeister, A C Nugent, D S Charney, W C Drevets, G Hasler

**Affiliations:** 1 Division of Molecular Psychiatry, Translational Research Center, University Hospital of Psychiatry, University of Bern, Bern, Switzerland; 2 Molecular Imaging Program, Department of Psychiatry and Radiology, New York University School of Medicine, New York, NY, USA; 3 Experimental Therapeutics & Pathophysiology Branch, Intramural Research Program, National Institute of Mental Health, National Institutes of Health, and Department of Health and Human Services, Bethesda, MD, USA; 4 Department of Psychiatry, Icahn School of Medicine at Mount Sinai, New York, NY, USA; 5 Laureate Institute for Brain Research, Tulsa, OK, USA; 6 Janssen Pharmaceuticals Research & Development, Titusville, NJ, USA

## Abstract

Despite immense efforts into development of new antidepressant drugs, the increases of serotoninergic and catecholaminergic neurotransmission have remained the two major pharmacodynamic principles of current drug treatments for depression. Consequently, psychopathological or biological markers that predict response to drugs that selectively increase serotonin and/or catecholamine neurotransmission hold the potential to optimize the prescriber's selection among currently available treatment options. The aim of this study was to elucidate the differential symptomatology and neurophysiology in response to reductions in serotonergic versus catecholaminergic neurotransmission in subjects at high risk of depression recurrence. Using identical neuroimaging procedures with [^18^F] fluorodeoxyglucose positron emission tomography after tryptophan depletion (TD) and catecholamine depletion (CD), subjects with remitted depression were compared with healthy controls in a double-blind, randomized, crossover design. Although TD induced significantly more depressed mood, sadness and hopelessness than CD, CD induced more inactivity, concentration difficulties, lassitude and somatic anxiety than TD. CD specifically increased glucose metabolism in the bilateral ventral striatum and decreased glucose metabolism in the bilateral orbitofrontal cortex, whereas TD specifically increased metabolism in the right prefrontal cortex and the posterior cingulate cortex. Although we found direct associations between changes in brain metabolism and induced depressive symptoms following CD, the relationship between neural activity and symptoms was less clear after TD. In conclusion, this study showed that serotonin and catecholamines have common and differential roles in the pathophysiology of depression.

## Introduction

Almost all available antidepressants bring about their effects by increasing monoamine neurotransmission, and many drugs that increase monoamines in the synaptic cleft have been shown to have antidepressant properties.^1^ Despite the considerable noise in placebo-controlled clinical trials, such trials showed a statistically significant advantage for switching patients with selective serotonin reuptake inhibitor (SSRI)-resistant depression to a non-SSRI rather than another SSRI antidepressant,^2^ which suggests important interindividual variation in the response to specific monoaminergic drugs. As a result, biomarkers predicting outcomes of specific monoaminergic drug classes have the potential to reduce the current trial-and-error method that commonly delays effective treatment. However, until now no such marker has been consistently identified for any monoaminergic antidepressant class. Thus, studies are needed that build a framework for guiding the selective, personalized antidepressant therapy by relating clinical symptoms and brain circuitry responses to serotoninergic and catecholaminergic neurotransmission.

In this study, we applied tryptophan depletion (TD) and catecholamine depletion (CD) to elucidate the common and differential symptoms and regional cerebral glucose metabolic changes these challenges induce in subjects with a history of major depressive disorder (MDD). The study compared data acquired in two previously published experiments from the same laboratory that used the identical neuroimaging procedure with positron emission tomography (PET) and [^18^F] fluorodeoxyglucose (^18^FDG) after TD or CD in subjects with fully remitted MDD (rMDD). TD, which putatively lowers central serotonergic transmission, was induced by depleting the serotonin precursor, tryptophan, through oral loading with all essential amino acids, except tryptophan. CD, which is expected to reduce central dopamine and norepinephrine neurotransmission, was achieved by administering α-methyl-paratyrosine (AMPT),^3^ a competitive inhibitor of tyrosine hydroxylase, the rate-limiting enzyme in the synthesis of catecholamines.^4^ CD has been shown to induce depressive symptoms in a relatively high proportion of subjects with rMDD, but generally does not affect mood in healthy controls.^5^

On the basis of previous studies suggesting functional interactions between serotonin and norepinephrine neurons in animal models of depression,^6, 7, 8^ reductions of serotoninergic and catecholaminergic neurotransmission by means of TD and CD are likely to induce both common and distinct effects on the spectrum of depressive symptoms.^3, 6, 9, 10, 11, 12, 13, 14, 15, 16, 17, 18, 19^

Previous neuroanatomical findings of serotonin,^20^ norepinephrine^21^ and dopamine^22^ have suggested that monoaminergic neurotransmission is involved in a wide range of cerebral functions including cognition, attention, mood, reward processing, appetite and sleep. As a result, deficiency of monoamines may conceivably explain the wide range of depressive symptoms including cognitive dysfunction, depressed mood and appetite and sleep disturbances. Functional neuroimaging studies have associated the reductions of specific monoamines with changes in hemodynamic or metabolic activity in distinct cerebral networks.^23^ The limbic–cortical–striatal–pallidal–thalamic network is of particular interest. This network connects the orbitofrontal cortex (OFC), medial prefrontal cortex (PFC), amygdala, hippocampus, ventromedial striatum, ventral pallidum and the mediodorsal and midline thalamic nuclei^23, 24, 25, 26^ that showed altered neurotransmission under the depletion of serotonin^27^ and catecholamines.^28^ In an exploratory fashion, the current study investigated how this altered neurotransmission differed quantitatively between TD and CD, and how these differences related to the type and severity of induced symptoms.

## Materials and methods

The current study compared two previously published experiments. The first study applied TD,^27^ the second study used CD in subject samples selected via the same entrance criteria.^28^

### Participants

Both studies (TD and CD) used the identical study procedure with respect to the study design and participant selection, that is, a double-blind, placebo-controlled crossover design in fully remitted, unmedicated depressed patients (rMDD). TD compared the effects of TD versus placebo and CD compared the effects of CD versus placebo. During the depletion procedures, the cerebral glucose metabolism was measured by PET and ^18^FDG. The experimental group in both studies comprised individuals aged 18–56 years who met DSM-IV criteria for MDD in full remission (rMDD). The healthy controls had no history of any psychiatric disorder and no major psychiatric disorder in first-degree relatives. Diagnosis was established by the Structured Clinical Interview for DSM-IV^29^ and confirmed by an unstructured interview with a psychiatrist. The subjects were recruited through the outpatient clinical services of the National Institute of Mental Health and by advertisements in local newspapers and posters on the National Institutes of Health campus. Exclusion criteria included major medical illnesses, pregnancy, psychotropic drug exposure (including nicotine) within 3 months, substance abuse within 1 year, lifetime history of substance dependence, psychiatric disorders other than MDD and structural brain abnormalities on magnetic resonance imaging (MRI). Inclusion criteria required that rMDD subjects had remained in remission without medications for at least 3 months and had manifested depression onset before 40 years of age. Written informed consent was obtained as approved by the institutional review board of the National Institute of Mental Health. With the exception of two rMDD subjects and five healthy controls who participated in both studies, the TD and CD study comprised independent subject samples. The TD study included 28 rMDD subjects (19 women, 9 men) and 27 healthy controls (18 women, 9 men). The CD study comprised 17 rMDD subjects (16 women, 1 man) and 13 healthy controls (12 women, 1 man). There were significantly more men in the rMDD group in the TD compared with the CD study (*P*=0.04). No PET data were obtained in one subject with rMDD and one healthy control subject in the TD study, and in two subjects with rMDD in the CD study.

### Tryptophan depletion

Subjects underwent two identical sessions that were separated by at least 8 days to avoid carryover effects. TD was induced by administration of 70 white capsules containing an amino-acid mixture consisting of isoleucine (4.2 g), leucine (6.6 g), lysine (4.8 g), methionine (1.5 g), phenylalanine (6.6 g), threonine (3.0 g) and valine (4.8 g) at 0700 hours (see Neumeister *et al.*^27^ for details). Placebo administration at 0700 hours comprised 70 white capsules with 31.5 g of lactose. Patients were restricted from eating upon completion of PET at about 1600 hours on day 1. Behavioral measures included the Hamilton Scale of Depression (HAMD), the Montgomery–Åsberg Depression Rating Scale (MADRS) and the Beck Anxiety Inventory (BAI). Study raters were blinded.

### Catecholamine depletion

Subjects underwent two identical sessions separated by at least 1 week, in which they received either a body-weight-adjusted AMPT dose or placebo (see Hasler *et al.*^28^ for details). To reduce risk of adverse reactions, a body-weight-adjusted AMPT dose of 40 mg kg^−1^ of body weight orally, to a maximum of 4 g, over 22 h was used. Each session took 3 days and was performed on an inpatient basis at the National Institutes of Health Clinical Center. To reduce the risk of crystalluria during AMPT administration, subjects received sodium bicarbonate, drank at least 2 l of water daily and underwent urinalysis twice daily. Behavioral measures included the HAMD, the MADRS and the BAI. Study raters were blinded.

### Statistical analysis of behavioral data

To compare the depletion effects of TD and CD on behavioral measures, differences in behavioral measures (ΔHAMD, ΔMADRS and ΔBAI) between challenge and placebo were calculated first for each subject and time point. These behavioral differences (ΔHAMD, ΔMADRS and ΔBAI) were then modeled with full factorial linear mixed models with restricted maximum likelihood estimations to account for the repeated measurements in the same subjects. Schwarz's Bayesian criteria were used to determine the best fitting covariance structure for each set of measures in cases where the typical compound symmetry approach used by analysis of variance did not provide the appropriate structure for the data. The effects of depletion type, diagnosis, depletion type-by-diagnosis and time on the ΔHAMD, ΔMADRS and ΔBAI scores were assessed with linear mixed models with an autoregressive covariance structure. Subject number and depletion sequence were included as random effects in all models. In addition, the factor gender was included in all models to regress-out this possible confounder since there were significantly more male subjects in the TD study. Furthermore, a *post hoc* analysis involving only the females alone was performed to prove that the statistical analysis adequately controlled for the gender difference. *Post hoc* *t*-tests involved a Tukey correction for multiple comparisons. Additional analyses assessed the different items measured with the HAMD, the MADRS and the BAI in detail using *t*-tests in rMDD to test for differences between TD and CD across symptom dimensions. The significance thresholds for these contrasts were set at alpha=0.05, two tailed. SAS 9.3 (SAS Institute, Cary, NC, USA) was used for all analyses. The means of the data are reported with their associated s.e.

### PET imaging

The PET imaging methods have been described in detail in our previous reports on the same participant cohort.^27, 28^ Both studies used the same procedures with respect to PET imaging. The PET images were acquired when the peak behavioral response was expected, that is, in the TD study, PET was measured 6 h after the administration of the amino-acid mixture/placebo, because the peak effects were expected at 5–7 h; in the CD study, PET images were acquired 30 h after the administration of the first AMPT/placebo dose, which corresponded to the time period when peak behavioral response was expected.^3^ Scanning was performed with a GE Advance scanner in three-dimensional mode (35 contiguous slices, 4.25 mm thick; three-dimensional resolution=6 mm full-width at half-maximum; GE Healthcare, Waukesha, WI, USA) and a slow bolus (over 2 min) injection of ^18^FDG. In order to obviate the need for arterial blood sampling, cerebral glucose utilization was quantified using a method that combines the left ventricular chamber time–tissue radioactivity data that were measured with dynamic PET imaging of the heart with venous blood sampling in order to provide ^18^FDG input function.^30^ This method has been validated previously by comparing it to more invasive approaches that use arterial plasma sampling.^30^ During image processing, the left ventricular time–radioactivity curve was extended in time to include the time of the brain emission scan by obtaining venous blood samples 25, 30, 35 and 50 min after the ^18^FDG injection. The mean radioactivity of these samples was divided by the mean left ventricular radioactivity concentration between 25 and 35 min post injection. This ratio was used to scale the 50-min venous sample concentration, which then was appended to the left ventricular curve in order to complete the input function that was used to generate parametric images of the regional cerebral metabolic rates for glucose (rCMRglu), as described by Moore *et al.*^30^ To provide an anatomical framework for the analysis of the PET images, structural MRI scans were acquired with a 3.0-T scanner (Signa; GE Medical Systems, Waukesha, WI, USA) applying a three-dimensional magnetization-prepared rapid acquisition gradient-echo sequence (echo time 2.982 ms; repetition time 7.5 ms; inversion time 725 ms; voxel size 0.9 × 0.9 × 1.2 mm).

### PET imaging: region-of-interest analysis

To compare the effects of TD versus placebo with CD versus placebo on cerebral metabolism, first a region of interest (ROI)-based analysis (with *P*-values corrected for the number of ROIs), and then a voxelwise analysis (with *P*-values corrected for the number of independent comparisons across the entire brain) were performed. For the ROI analysis, MEDx (Medical Numerics, Sterling, VA, USA) software was used. ROIs were selected according to previous results in rMDD^5, 31^ and untreated, symptomatic patients with MDD,^32^ which showed alterations in the OFC, posterior cingulate cortex (PCC), medial thalamus, and dorsolateral prefrontal cortex (DLPFC), ventral striatum, anterior PFC, pregenual PFC, subgenual PFC, ventrolateral PFC, anteromedial PFC, amygdala, hippocampus and anterior insula. The ROIs were defined *a priori* on an MRI template and were placed on each patient's registered MRI, using anatomical definitions described previously.^27^ A binary mask of the gray matter was used to restrict all further analyses to gray matter voxels. To account for nonspecific global effects, the whole-brain metabolism was used to normalize the regional measures (the quantitative measures of whole-brain metabolism obtained under each depletion type revealed no significant difference in global metabolism for either CD or TD, as reported previously^27, 28^). The normalized mean metabolic activity was then obtained for each ROI in each subject and each session, and the regional differences (ΔrCMRglu) between sessions (TD versus placebo and CD versus placebo) were calculated for each subject. The statistical models that were applied to compare the ΔrCMRglu in each ROI included the main effects of depletion, diagnosis and their interaction. The significance level was Bonferroni corrected for the number of 13 ROIs. The significance threshold was set at alpha=0.05, two tailed. All *P*-values are reported before correction for multiple comparisons.

### PET imaging: voxelwise analysis

For the whole-brain analyses, we used Matlab (Matlab version 8, release 14; The MathWorks, Natick, MA, USA), SPM8 (Wellcome Trust Centre for Imaging, London, England; http://www.fil.ion.ucl.ac.uk/spm/software/spm8), and the toolbox aslm.^33^ PET images were coregistered to the MRIs and spatially normalized to the Montreal Neurological Institute brain template with SPM8. Images were filtered with a 6-mm Gaussian smoothing kernel in order to compensate for interindividual anatomical variability. The statistical analysis of whole-brain metabolism involved a flexible factorial model in SPM8 with the factors depletion, diagnosis and subject. Two additional regressors were included to account for the oversampling of female subjects in the CD study and for the nonspecific fluctuations in the whole-brain metabolism. Clusters with a voxel-level threshold of *P*<0.05, whole-brain corrected for false discovery rate are reported for regions without *a priori* hypotheses.

### PET imaging: correlational analysis

Depression and anxiety items showing significant differences between TD and CD were analyzed in an exploratory *post hoc* analysis. Spearman rank correlations were calculated that assessed associations between those items and metabolism in the ROIs in rMDD subjects. The significance threshold was set at alpha=0.05, two tailed.

## Results

The clinical and demographic characteristics of the subject samples are detailed in Table 1.

### Behavioral effects of TD compared with CD

Both TD and CD induced more depressive symptoms in rMDD subjects compared with controls, but the depletion effect of TD compared with CD did not differ significantly as measured on either the HAMD (*P*=0.37) or the MADRS (*P*=0.27). In addition, there was no depletion type (TD versus CD)-by-diagnosis interaction on either scale's total score. Furthermore, TD and CD induced more anxiety symptoms as assessed using the BAI in subjects with rMDD compared with controls, but the effect did not significantly differ between depletion types (*P*=0.14). There was no depletion type-by-diagnosis interaction evident on the change in anxiety ratings. Repeating the analyses in female subjects alone did not alter the results.

To assess the between-subject variation between the TD and CD study, we also calculated one-factor analyses of variance with the factor depletion type (two levels; TD and CD) and the dependent variables MADRS and BAI, respectively, including all measures during the placebo conditions. There were no significant differences in between-subject variations in MADRS scores (F(1,167)=2.53, *P*=0.11) and BAI scores (F(1,166)=0.42, *P*=0.52), respectively.

### Detailed analysis of HAMD and MADRS items in rMDD subjects

**Figure 1:**
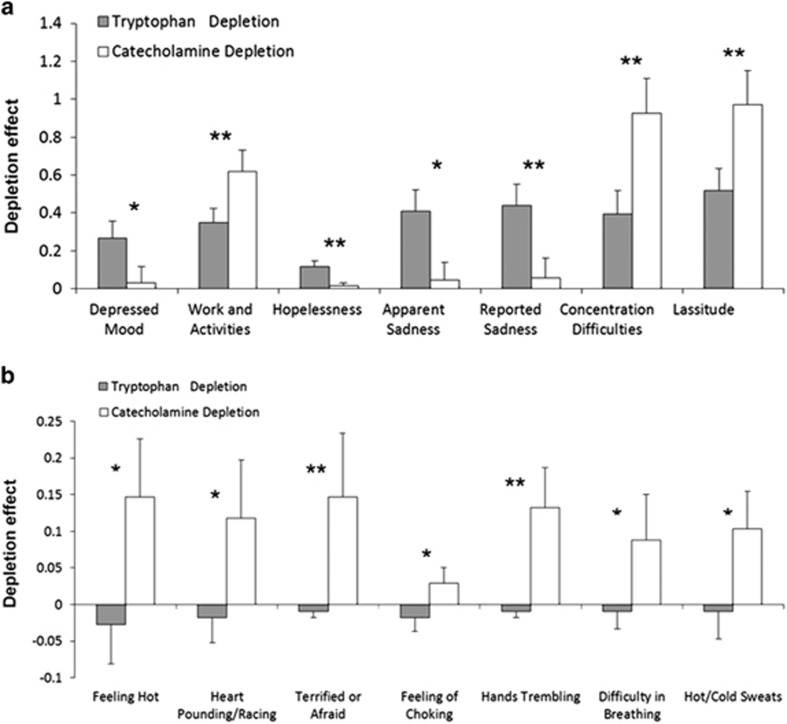
Depression and anxiety items showing significant differences in the tryptophan depletion effect compared with the catecholamine depletion effect in remitted major depressive disorder subjects are displayed with means and s.e. Items were sampled using the Hamilton Scale of Depression and Montgomery–Åsberg Depression Rating Scale (**a**) and the Beck Anxiety Inventory (**b**). Significant at *P<0.05; significant at **P<0.01.

Individual items from the HAMD and the MADRS showing significant differences in the TD effect compared with the CD effect in rMDD are displayed in Figure 1a. Compared with CD, TD induced stronger effects on depressed mood (*t* (1,254) =2.52, *P*=0.01), hopelessness (*t* (1,252)=3.15, *P*=0.002), apparent sadness (*t* (1,254)=2.56, *P*=0.01) and reported sadness (*t* (1,254)=3.07, *P*=0.002). The depletion effect of CD was stronger compared with TD on work and activities (*t* (1,254)=2.85, *P*=0.005), concentration difficulties (*t* (1,254)=2.92, *P*=0.004) and lassitude (*t* (1,252)=2.89, *P*=0.004).

Repeating the analyses in female subjects only weakened the stronger effects of TD compared with CD on depressed mood (*t* (1,194)=1.77, *P*=0.08) and apparent sadness (*t* (1,194)=1.93, *P*=0.06), potentially due to the reduction in sample size. In addition, TD had stronger effects on hypochondriasis (*t* (1,194)=2.21, *P*=0.03) and CD had stronger effects on the inability to feel (*t* (1,194)=2.01, *P*<0.05) in females. The results for the other items remained unchanged between the entire-group comparison versus the females-alone comparison.

### Detailed analysis of BAI items in rMDD subjects

Anxiety items showing significant differences in the TD effect compared with the CD effect in rMDD are displayed in Figure 1b. Compared with the TD condition, CD induced significantly greater feelings of flushing (*t* (1,248)=2.44, *P*=0.02), palpitations (*t* (1,248)=2.07, *P*=0.04), fear (*t* (1,248)=3.38, *P*=0.0008), choking (*t* (1,248)=2.55, *P*=0.01), tremulousness (*t* (1,248)=3.03, *P*=0.003), dyspnea (*t* (1,247)=2.39, *P*=0.02) and diaphoresis (*t* (1,248)=2.05, *P*=0.04). In the analyses limited to female subjects, no significant difference was found on either the palpitations or the diaphoresis items, but the results on the other items were similar to those found in the entire sample.

### ROI analyis of PET data

**Figure 2:**
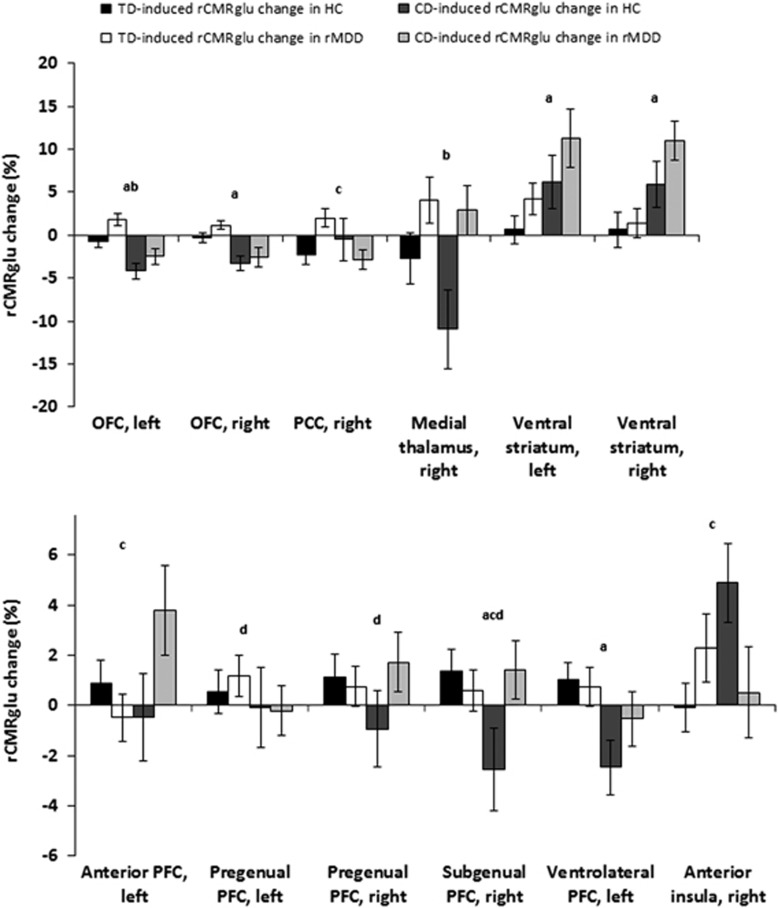
The mean percent change (with s.e.) in normalized regional glucose metabolism induced by tryptophan depletion (TD) and catecholamine depletion (CD) in *a priori* defined regions of interest (ROIs) in subjects with remitted major depressive disorder (rMDD) and healthy controls (HCs). Normalized values were obtained by dividing each mean value by the corresponding whole-brain glucose metabolism value to factor out nonspecific global effects. Significant depletion effect at *P*<0.05: a; significant diagnosis effect at *P*<0.05: b; significant depletion-by-diagnosis interaction at *P*<0.05: c; significant sex effect at *P*<0.05: d. OFC, orbitofrontal cortex; PCC, posterior cingulate cortex; PFC, prefrontal cortex; rCMRglu, regional cerebral metabolic rates for glucose.

No difference was found between TD- and CD-induced effects on mean whole-brain glucose metabolism (*P*=0.26). Figure 2 shows *a priori* defined ROIs that showed a significant difference in the TD compared with the CD effect on normalized regional metabolism. In the OFC there was a decrease in metabolism induced by CD compared with TD across groups (left: F (1,76)=20.7, *P*<0.0001; right: F (1,76)=17.4, *P*<0.0001). In addition, glucose metabolism in the left OFC in rMDD subjects was higher than in healthy controls (F (1,76)=7.30, *P*=0.009). In the right PCC, there was a significant depletion type-by-diagnosis interaction (F (1,74)=6.99, *P*=0.01) that was attributable to a higher TD effect than CD effect in rMDD and higher metabolism induced by TD in rMDD compared with controls. The right medial thalamus showed increased metabolism in rMDD compared with controls across studies (F (1,68)=8.58, *P*=0.005). The CD-induced increase in metabolism in the ventral striatum was higher than the increase under TD (left: F (1,76)=5.53, *P*=0.02; right: F (1,76)=10, *P*=0.002). There was a significant depletion type-by-diagnosis interaction on the regional glucose metabolism of the left anterior PFC (F (1,76)=4.82, *P*=0.03) that was attributable to a CD-induced metabolic increase compared with a TD-induced metabolic decrease in rMDD subjects. In the pregenual PFC, female subjects showed a significant higher metabolism compared with male subjects across studies and groups (left: F (1,76)=5.8, *P*=0.02; right: F (1,76)=6.61, *P*=0.01). The right subgenual PFC showed a higher metabolism across groups induced by TD (F (1,76)=3.98, *P*<0.05) and a significant depletion type-by-diagnosis interaction (F (1,76)=4.78, *P*=0.03) that was attributable to a higher metabolism induced by TD compared with CD in controls; in addition, female subjects showed a higher metabolism in this region compared with male subjects across studies and groups (F (1,76)=4.42, *P*=0.04). The left ventrolateral PFC showed a TD-induced increase in metabolism across groups compared with a decrease in the CD study (F (1,76)=5.26, *P*=0.02). In the right anterior insula, there was a significant depletion type-by-diagnosis interaction (F (1,76)=5.26, *P*=0.02) that was attributable to a decreased metabolism induced by CD in rMDD subjects compared with controls and an increased metabolism induced by CD compared with TD in controls. After applying Bonferroni corrections, the effects in the right and left OFC and in the right ventral striatum remained significant. After repeating the analyses in female subjects alone, the depletion type-by-diagnosis interaction in the left anterior PFC was reduced to a nonsignificant trend (*P*=0.13); the depletion type-by-diagnosis interaction in the right anteromedial PFC (*P*=0.08) and the right subgenual PFC (*P*=0.06) and the effect of depletion type on the left ventrolateral PFC (*P*=0.06) remained as trend effects. In females only, metabolism was higher after CD compared with TD across groups in the right anterior PFC (F (1,57)=11.92, *P*=0.001) and in the right hippocampus (F (1,57)=5.05, *P*=0.03), and higher after TD compared with CD across groups in the PCC (left: F (1,57)=4.27, *P*=0.04; right: F (1,55)=6.31, *P*=0.02). All other results remained essentially unchanged from the comparisons involving the entire group with respect to statistical significance.

### Voxelwise analysis of PET data

A depletion type-by-diagnosis interaction was evident in the right lingual gyrus that survived applying correction for false discovery rate (peak: *t* (1,83)=4.65, size=34 voxel, *P*<0.05, Brodmann area 18). *Post hoc* tests showed this interaction was attributable to a significant decrease in regional metabolism under CD in the rMDD subjects (*t* (1,54)=2.04, *P*<0.05) but not in the controls (*P*=0.68), and no change under TD in either the rMDD subjects (*P*=0.25) or the controls (*P*=0.56).

### Correlational analysis of depression and anxiety items with PET data

Table 2 shows Spearman rank correlations (rho) of depression and anxiety symptoms where significant differences between TD and CD had been found, and the corresponding regional glucose metabolism changes in the ROIs. As there were no significant correlations of TD-induced changes in depression and anxiety symptoms with corresponding changes in regional glucose metabolism, all values correspond to findings that were induced by CD. After applying Bonferroni corrections, only the correlation between the apparent sadness and the left anterior PFC remained significant. We also assessed how the relationship between changes in specific depression and anxiety items and changes in regional glucose metabolism were moderated by the depletion type and thus the differences in neurotransmitter levels. Results can be found in the Supplementary Information (Supplementary Table S1). In addition, we compared the significant correlation coefficients with the corresponding coefficients of the ROI in the complementary hemisphere; results can be found in the Supplementary Information (Supplementary Table S2).

## Discussion

We believe the current study is the first to compare the behavioral and neural effects of TD versus CD in unmedicated rMDD subjects and healthy controls. These challenges putatively reflected depletions in serotoninergic and catecholaminergic neurotransmission, respectively, which are of interest in patients with MDD because currently available antidepressant treatments enhance the function of one or both of these systems. Table 3 displays the main findings of this study. Although brain activity was correlated with distinct depressive symptoms following CD, there was no direct relationship between specific symptoms and brain activity following TD.

The behavioral and neural effects of CD and TD showed some shared effects that are compatible with the literature.^3, 6, 9, 10, 11, 12, 13, 14, 15, 16, 17, 18^ However, the different study design of the current study has to be considered as it was conducted in fully rMDD patients. Both depletion methods induced depressive symptoms as measured using the HAMD and MADRS, and both increased cerebral glucose metabolism in the medial thalamus, the OFC and the ventral striatum. These findings suggest that interactions between the monoamine systems are involved in the pathogenesis of depression. The medial thalamus has an important relay function in connecting sensory and basal ganglia inputs with prefrontal cortical structures. The abundant serotoninergic and dopaminergic innervation of the thalamus has been shown to participate in complex synergistic or opposing interactions, potentially contributing to the similar impact of TD and CD on thalamic glucose metabolism, and conceivably on several depressive symptoms. For example, the thalamus receives serotonergic afferents from the dorsal and median raphe nucleus,^34, 35^ which participate in the neural processing underlying anxiety-related behaviors^36, 37^ and the generation of various stages of the sleep–wake cycle.^38^ In addition, the dopaminergic system modulates neural transmission within the limbic–thalamo–cortical circuits that involve regions of the medial and orbital prefrontal cortex, ventral striatum and amygdala, which modulate reward-related learning and motivation.^39^

Dysfunction of this pathway may underlie a range of depressive symptoms including lack of motivation, including problems related to work and activities. For example, the OFC contains dopaminergic terminals and receptors^40^ and blockade of these receptors reduces the break point of rats responding on a progressive ratio schedule of reinforcement, a classic test of incentive motivation.^41^ In addition, dopamine depletion in the OFC impaired responding for delayed reward.^42^ Moreover, the OFC also receives serotonergic innervation from the dorsal and median raphe nuclei, and the reciprocal innervation from the OFC enables the OFC to regulate not only its own serotonin input but the serotonin input to the rest of the forebrain, which has been associated with the capability of animals to adapt to changing reward contingencies.^43^ Notably, depletion of serotonin has been shown to impair this flexibility of the reward system.^43^ In addition, it has been suggested that serotonin and dopamine modulate different functions in the OFC with orbitofrontal serotonin preventing competing, task-irrelevant stimuli from biasing task-based responding, processes that may hold relevance for the attentional biases toward negative stimuli extant in MDD.^44, 45^ Our results further appear in line with the clinical observation that motivational deficits and the inability to concentrate are closely connected.^46^

TD more specifically induced symptoms of sadness and depressed mood. Serotonin has a well-known function in the emotional inhibition and regulation, and acute TD has been shown to induce a negative attentional and mnemonic bias both in rMDD subjects and in healthy controls.^47, 48, 49^ In the right PCC, TD compared with CD induced a metabolic increase compared with a decrease in rMDD and a significant difference between rMDD and controls in TD only. The PCC has a specific role in the regulation of pain, which has been related theoretically to negative effect. Consequently, serotonin deficiency may both increase negative emotion and reduce emotional control. In addition, given that the PCC is a critical node in serotonin neurotransmission^50^ and is also implicated in self-referential processes as a hub of the default mode network, this study adds to the evidence that serotonin deficiency has an important role in default mode network overactivity in depression.^51^ Nevertheless, the correlation between TD-induced changes in regional metabolism and TD-induced depressive symptoms was not significant, suggesting that the relationship between serotonin, brain metabolism and sad/depressed mood is complex.

CD specifically induced symptoms of reduced activity, impaired concentration and lassitude and specifically increased brain metabolism in specific PFC regions and the ventral striatum. Dopamine depletion in monkeys leads to cognitive and attention deficits in the primate PFC,^52, 53^ potentially resembling the concentration problems encountered in some depressed humans. This is supported by our study that found that the CD-induced increase in brain metabolism in the anterior PFC was correlated with concentration difficulties induced by CD. The findings of increased lassitude and of work and activity problems conceivably support hypotheses^54, 55^ that a dopamine deficit underlies dysfunctional reward processing, which leads to amotivation-related symptoms in MDD. As shown in Table 2, the CD-induced increase in brain metabolism in the striatum was directly associated with lassitude, and the CD-induced increase in brain metabolism in the ACC was associated with self-reported sadness. This argues for a relatively direct mechanistic relationship between lack of dopamine and/or norepinephrine in the pathogenesis of distinct depressive symptoms.

In the left anterior PFC, the glucose metabolism increased under CD but decreased under TD. The anterior PFC has been linked to the processing of affective salience of sensory stimuli in previous studies,^56, 57^ which may relate to reduced activity and lassitude depressive symptoms. However, this effect was weakened to a trend level when repeating the analysis in female subjects only.

One of the most consistent functional imaging findings in MDD is increased metabolism in the subgenual and ventrolateral PFC.^58, 59, 60^ We found metabolic increases in these brain regions to be more pronounced under TD compared with CD, suggesting that serotonin deficiency is more important in the pathogenesis of the hyperactivity of these brain regions that are both part of the extended visceromotor networks, which participates in regulating the neuroendocrine, autonomic and experiential aspects of emotion.^24^

We found some interhemispheric differences in our correlational analysis of CD-induced changes in mood and neurotransmission, with the most prominent being the positive correlation between changes in depressed mood and changes in regional glucose metabolism in the left DLPFC and a corresponding negative correlation in the right DLPFC. Interhemispheric differences in the DLPFC have been reported in several studies measuring resting state^25, 61^ and have been the neurobiological basis for therapeutic brain stimulation paradigms with transcranial magnetic stimulation^62^ and transcranial direct current stimulation.^63^ Specifically, hypoactivity in the left DLPFC has been linked to negative emotional judgment and hyperactivity in the right DLPFC to attentional modulation.^64^ Our findings suggest that interhemispheric differences in the DLPFC in depression are related to a deficit in catecholaminergic neurotransmission.

Unexpectedly, the voxelwise analysis identified reduced glucose metabolism under CD in the right lingual gyrus in the unmedicated rMDD sample. Reductions in this region have been previously reported in a study in young MDD adults^65^ and abnormal focal magnetic low-frequency activity has been found in untreated patients with MDD.^66^ The current study adds that abnormality in this brain region is associated with reduced catecholaminergic neurotransmission.

Interestingly, no difference was found in the current study between TD and CD on global levels of anxiety, as measured with the BAI. We found stronger effects of CD compared with TD on several anxiety items including greater feelings of flushing, palpitations, fear, choking, tremulousness, dyspnea and diaphoresis. A possible explanation for this observation is that somatic anxiety symptoms in response to threat and stress are modulated more potently by central catecholaminergic pathways than via central serotonergic pathways.^67^

The current study had several strengths that are noteworthy. First, it compared two experiments that took place at the same scanning site and used the same PET imaging procedure for both the TD and the CD studies. Second, the fact that a sample of subjects with rMDD off medication was assessed allowed us to investigate behavioral and neural effects of serotonin- and catecholamine-related pathways unbiased by medication. Further, we could interpret the findings as risk factors for a depressive relapse. Finally, in contrast to our findings, previous studies involving SSRI and norepinephrine reuptake inhibitor pharmacotherapy of MDD patients found surprisingly small differences between serotoninergic and catecholaminergic agents on depressive symptoms.^68^ Possible reasons for this discrepancy could be the insufficient specificity of chronic SSRI and norepinephrine reuptake inhibitor administration, as the 6–8-week duration of therapeutic trials allows for adaptive changes to occur in other neurotransmitter systems and for a placebo effects to increase, which both may blur differences across challenges.^69^ The shorter time frame needed for comparing the effects of acute TD versus acute CD thus may offer greater sensitivity than clinical trials using monoaminergic antidepressant drugs, because the acute nature of both depletion methods ensures a higher specificity for serotonin and catecholamine systems, respectively, along with relatively smaller placebo effects. In future studies, refining our approach using a placebo-controlled, double-blind, crossover design would enable within-subject comparisons with increased power to detect differences between depletion paradigms.

The current study had several limitations that merit comment. The study sample was relatively small and contained a majority of female subjects. We took this into account by including a regressor for gender in each analysis and by repeating all analyses with female subjects only. In addition, PET imaging of glucose metabolism did not specifically assess central serotonin or catecholamine concentrations. Instead, we assessed the central effect of TD indirectly by measuring plasma total and free tryptophan levels and the central effect of CD by assessing serum prolactin levels, which is the standard method to assess the effect of central CD.^28, 70^ A preferable design would have been a within-subjects design, whereby all participants underwent both depletion procedures. However, the two experiments took place at the same scanning site and used the same PET imaging procedure for both the TD and the CD studies, increasing the comparability of the two studies. In addition, an analysis of between-subject variance between TD and CD during the placebo condition did not reveal any significant differences. Finally, a noninvasive method for comparing the depth of CD versus TD within the brain is not available. Given the similar amount of depressive symptoms induced by both methods and the similar effect of both methods on metabolism in at least some brain structures, we assumed that TD and CD, as used in our study, were comparable regarding the hypotheses we aimed to test.

Taken together, these data suggest that serotonin and catecholamines have both common and distinct roles in the neurobiology of depressive symptoms. This study further suggests that the development of psychopathological and neuronal markers predicting response to selective monoamine inhibition may be feasible. Finally, this study provides a rationale for the use of antidepressants with primary pharmacological actions involving both serotonergic and catecholaminergic mechanisms in some patients.^71^

## Figures and Tables

**Table 1:** Demographic and clinical characteristics of unmedicated subjects with remitted major depressive disorder (rMDD) and healthy controls

*Characteristic*	*TD (*n=*55)*		*CD (*n=*30)*	
	*HC*	*rMDD*	*HC*	*rMDD*
Sex no., f/m	18/9	19/9	12/1	16/1
Age, mean (s.d.), years	34.2 (11.2)	39.8 (12.7)	39.1 (9.6)	39.2 (10.8)
MADRS at study entry	0.6 (1.2)	1.4 (1.8)	0.4 (0.9)	1.6 (1.9)
HAMD at study entry	0.9 (1.1)	1.3 (1.4)	0.4 (0.8)	1.6 (1.1)
BAI at study entry	0.8 (1.2)	2.3 (2.6)	0.5 (1.1)	1.9 (1.7)

Abbreviations: BAI, Beck Anxiety Inventory; CD, catecholamine depletion; f/m, female/male; HAMD, Hamilton Depression Scale; HC, healthy control; MADRS, Montgomery–Åsberg Depression Rating Scale; NA, not applicable; no., number; TD, tryptophan depletion.

**Table 2:** Spearman rank correlations (rho) of changes in depression and anxiety symptoms with changes in regional glucose metabolism

	*Apparent sadness*	*Reported sadness*	*Concentration difficulties*	*Lassitude*	*Depressed mood*	*Work and activities*	*Feeling hot*	*Heart pounding/racing*	*Feeling of choking*	*Hands trembling*
DLPFC, left	0.17	0.07	0.03	**−**0.50	0.57*	−0.55*	0.02	**−**0.05	0.18	**−**0.37
DLPFC, right	−0.66**	−0.57*	**−**0.34	**−**0.09	−0.63*	**−**0.44	0.22	**−**0.11	**−**0.14	**−**0.30
Anterior PFC, left	0.79***	0.73**	0.71**	0.24	0.45	0.36	**−**0.46	**−**0.28	**−**0.50	**−**0.02
Anterior PFC, right	**−**0.06	**−**0.14	0.17	**−**0.02	0.02	**−**0.23	0.63*	**−**0.05	**−**0.23	**−**0.14
Hippocampus, left	**−**0.20	**−**0.19	**−**0.03	0.05	**−**0.07	**−**0.15	0.63*	**−**0.22	**−**0.27	**−**0.24
Hippocampus, right	0.50	0.46	0.70**	0.09	0.63*	0.09	**−**0.09	**−**0.27	**−**0.18	**−**0.11
Ventral striatum, left	0.53*	0.55*	0.19	−0.65**	0.58*	**−**0.19	0.03	0.09	0.05	**−**0.27
Ventral striatum, right	**−**0.31	**−**0.16	**−**0.41	−0.64*	**−**0.12	**−**0.09	0.25	**−**0.13	0.00	**−**0.32
Pregenual PFC, right	**−**0.34	**−**0.31	**−**0.12	**−**0.12	**−**0.31	0.00	**−**0.17	−0.53*	**−**0.41	**−**0.35
PCC, left	**−**0.37	**−**0.32	**−**0.42	**−**0.16	**−**0.32	0.08	0.29	0.49	0.14	0.55*
PCC, right	0.39	0.42	0.30	0.33	0.05	0.59*	**−**0.38	**−**0.13	**−**0.18	0.10
Anteromedial PFC, left	**−**0.02	**−**0.09	0.09	**−**0.01	0.07	**−**0.13	**−**0.27	0.49	0.54*	0.39
Anterior insula, left	0.28	0.23	**−**0.01	**−**0.22	0.59*	0.03	**−**0.01	0.20	0.23	0.10

Abbreviations: CD, catecholamine depletion; DLPFC, dorsolateral prefrontal cortex; PCC, posterior cingulate cortex; PFC, prefrontal cortex; TD, tryptophan depletion.

Items were chosen according to our previous analyses, that is, where we had found significant differences between TD and CD. Note that there were no significant correlations of TD-induced changes in depression and anxiety symptoms with corresponding changes in regional glucose metabolism, so all values correspond to findings that were induced by CD. Statistically significant correlations are indicated in bold. Significance at *P<0.05; significance at **P<0.01; significance at ***P<0.001.

**Table 3:** Summary of the main findings of the study, categorized by common and differential effects. Effects on behavior relate to subjects with remitted depression only

	*Common effects*	*Differential effects*	
		*TD>CD*	*CD>TD*
Behavior	Global HAMD Global MADRS Global BAI	Depressed mood Sadness	Work and activities Concentration difficulties Lassitude
Cerebral glucose metabolism	Whole brain Ventral striatum Medial thalamus, right OFC, left	Pregenual PFC, right Ventrolateral PFC, left PCC, right OFC	Anterior PFC, left Subgenual PFC, right

Abbreviations: BAI, Beck Anxiety Inventory; CD, catecholamine depletion; HAMD, Hamilton Depression Scale; MADRS, Montgomery–Åsberg Depression Rating Scale; OFC, orbitofrontal cortex; PCC, posterior cingulate cortex; PFC, prefrontal cortex; TD, tryptophan depletion.

## Notes

WCD is currently an employee of Janssen Pharmaceuticals of Johnson & Johnson. The remaining authors declare no conflict of interest.
