# Supplementary material for: Serotonin versus catecholamine deficiency: behavioral and neural effects of experimental depletion in remitted depression
Source: Transl Psychiatry. 2015 Mar 17;5(3):e532–. doi: 10.1038/tp.2015.25 (PMC4354355; doi:10.1038/tp.2015.25)
Supplement: Supplementary Information [file tp201525x1.doc]

# Supplementary information

# Table S1

We calculated additional general linear mixed models to assess how the relationship between changes in specific depression and anxiety items and changes in regional glucose metabolism were moderated by the depletion type and thus the differences in neurotransmitter levels. In this analysis, we included mood items (of MADRS, HAMD and BAI) and ROIs that had been shown to be differentially modulated by TD and CD in our previous analysis and that had shown a significant correlation in our correlational analysis (as summarized in Table 2). Results are presented in the following. We found that the correlations of symptom-change and change in regional metabolism was moderated by the type of depletion as reflected by the interaction term (metabolism*depletion type). Estimates of depletion type and metabolism effects as well as interaction terms are presented together with 95% confidence intervals (indicated as “Lower” and “Upper” in the table), F-values (with degrees of freedom, “DF1” and “DF2”), and P-values. In addition, “DT” indicates depletion type and is thought to show which of the depletions (TD, CD) was responsible for the corresponding effect. A positive value of the interaction term estimate (metabolism*depletion type) indicates the positive effect of an one-unit-increase of metabolism under the relevant depletion type on the change in the specific depression or anxiety item while a negative value indicates the corresponding negative effect.

# Table S2

We compared the correlation coefficients of CD-induced changes in mood and neurotransmission that had been shown to be statistically significant with the corresponding coefficients of the ROI in the complementary hemisphere. We therefore calculated Fisher-transformations of the correlations and computed a z-statistic to test for statistical significance. Z-values are presented, with bold values indicating statistical significance (p < 0.05). A positive value indicates that the correlation coefficient was significantly higher in the left hemisphere while a negative value indicates that the correlation coefficient was significantly higher in the right hemisphere. After a Bonferroni-correction, only the difference in DLPFC and depressed mood remained significant.

# Table S1

| **Item** | **ROI** | **Effect** | **DF1** | **DF2** | **F-val** | **P-val** | **DT** | **Estimate** | **Lower** | **Upper** |
| --- | --- | --- | --- | --- | --- | --- | --- | --- | --- | --- |
| Depressed mood | DLPFC, left | depletion type | 1 | 38 | 2.47 | 0.125 | CD | 0.30 | -0.09 | 0.68 |
|  |  | metabolism | 1 | 38 | 8.81 | 0.005 |  | 4.85 | -3.39 | 13.10 |
|  |  | metabolism*depletion type | 1 | 38 | 2.97 | 0.093 |  | 13.42 | -2.36 | 29.20 |
| Work and activities | DLPFC, left | depletion type | 1 | 38 | 43.79 | <.0001 | CD | 1.35 | 0.94 | 1.76 |
|  |  | metabolism | 1 | 38 | 6.69 | 0.014 |  | -2.13 | -11.00 | 6.74 |
|  |  | metabolism*depletion type | 1 | 38 | 4.32 | 0.045 | CD | -17.42 | -34.39 | -0.45 |
| Apparent sadness | DLPFC, right | depletion type | 1 | 38 | 0.92 | 0.343 | CD | 0.23 | -0.25 | 0.71 |
|  |  | metabolism | 1 | 38 | 4.14 | 0.049 |  | 7.19 | -1.69 | 16.06 |
|  |  | metabolism*depletion type | 1 | 38 | 21.46 | <.0001 | CD | -25.64 | -36.84 | -14.43 |
| Reported sadness | DLPFC, right | depletion type | 1 | 38 | 1.54 | 0.222 | CD | 0.27 | -0.17 | 0.72 |
|  |  | metabolism | 1 | 38 | 2.06 | 0.159 |  | 6.69 | -1.53 | 14.90 |
|  |  | metabolism*depletion type | 1 | 38 | 16.38 | 0.000 | CD | -20.73 | -31.10 | -10.36 |
| Depressed mood | DLPFC, right | depletion type | 1 | 38 | 3.48 | 0.070 | CD | 0.27 | -0.02 | 0.57 |
|  |  | metabolism | 1 | 38 | 11.68 | 0.002 |  | 1.65 | -3.79 | 7.10 |
|  |  | metabolism*depletion type | 1 | 38 | 19.29 | <.0001 | CD | -14.91 | -21.79 | -8.04 |
| Apparent sadness | Anterior PFC,  left | depletion type | 1 | 38 | 3.51 | 0.069 | CD | -0.45 | -0.94 | 0.04 |
|  |  | metabolism | 1 | 38 | 17.55 | 0.0002 |  | -1.64 | -7.12 | 3.84 |
|  |  | metabolism*depletion type | 1 | 38 | 25.56 | <.0001 | CD | 19.16 | 11.49 | 26.84 |
| Reported sadness | Anterior PFC,  left | depletion type | 1 | 38 | 1.47 | 0.233 | CD | -0.28 | -0.74 | 0.19 |
|  |  | metabolism | 1 | 38 | 13.97 | 0.001 |  | -0.54 | -5.68 | 4.60 |
|  |  | metabolism*depletion type | 1 | 38 | 16.33 | 0.000 | CD | 14.37 | 7.17 | 21.57 |
| Concentration difficulties | Anterior PFC,  left | depletion type | 1 | 38 | 58.7 | <.0001 | CD | 2.12 | 1.56 | 2.68 |
|  |  | metabolism | 1 | 38 | 10.17 | 0.003 |  | -2.08 | -8.34 | 4.18 |
|  |  | metabolism*depletion type | 1 | 38 | 17.22 | 0.000 | CD | 17.96 | 9.20 | 26.72 |
| Feeling hot | Anterior PFC, right | depletion type | 1 | 37 | 3.39 | 0.074 | CD | -0.27 | -0.57 | 0.03 |
|  |  | metabolism | 1 | 37 | 10.14 | 0.003 |  | -0.56 | -4.45 | 3.34 |
|  |  | metabolism*depletion type | 1 | 37 | 12.31 | 0.001 | CD | 12.07 | 5.10 | 19.04 |
|  |  |  |  |  |  |  |  |  |  |  |
| **Item** | **ROI** | **Effect** | **DF1** | **DF2** | **F-val** | **P-val** | **DT** | **Estimate** | **Lower** | **Upper** |
| Feeling hot | Hippocampus, left | depletion type | 1 | 37 | 0 | 0.958 | CD | -0.01 | -0.29 | 0.28 |
|  |  | metabolism | 1 | 37 | 9.51 | 0.004 |  | 0.52 | -1.73 | 2.77 |
|  |  | metabolism*depletion type | 1 | 37 | 5.64 | 0.023 | CD | 3.47 | 0.51 | 6.43 |
| Concentration difficulties | Hippocampus, right | depletion type | 1 | 38 | 43.39 | <.0001 | CD | 2.02 | 1.40 | 2.63 |
|  |  | metabolism | 1 | 38 | 16.53 | 0.0002 |  | 1.57 | -2.22 | 5.37 |
|  |  | metabolism*depletion type | 1 | 38 | 9.01 | 0.005 | CD | 8.88 | 2.89 | 14.87 |
| Depressed mood | Hippocampus, right | depletion type | 1 | 38 | 0.61 | 0.441 | CD | -0.16 | -0.56 | 0.25 |
|  |  | metabolism | 1 | 38 | 12.88 | 0.001 |  | 0.48 | -1.99 | 2.95 |
|  |  | metabolism*depletion type | 1 | 38 | 9.55 | 0.004 | CD | 5.95 | 2.05 | 9.85 |
| Apparent sadness | Ventral striatum, left | depletion type | 1 | 38 | 2.63 | 0.113 | CD | -0.57 | -1.28 | 0.14 |
|  |  | metabolism | 1 | 38 | 4.41 | 0.043 |  | -1.17 | -4.67 | 2.33 |
|  |  | metabolism*depletion type | 1 | 38 | 9.25 | 0.004 | CD | 7.56 | 2.53 | 12.59 |
| Reported sadness | Ventral striatum, left | depletion type | 1 | 38 | 1.53 | 0.224 | CD | -0.38 | -1.01 | 0.24 |
|  |  | metabolism | 1 | 38 | 3.01 | 0.091 |  | -1.28 | -4.35 | 1.80 |
|  |  | metabolism*depletion type | 1 | 38 | 8.45 | 0.006 | CD | 6.34 | 1.93 | 10.76 |
| Lassitude | Ventral striatum, left | depletion type | 1 | 38 | 52.73 | <.0001 | CD | 3.01 | 2.17 | 3.86 |
|  |  | metabolism | 1 | 38 | 7.13 | 0.011 |  | 0.03 | -4.11 | 4.16 |
|  |  | metabolism*depletion type | 1 | 38 | 7.22 | 0.011 | CD | -7.89 | -13.83 | -1.95 |
| Depressed mood | Ventral striatum, left | depletion type | 1 | 38 | 0.95 | 0.335 | CD | -0.22 | -0.68 | 0.24 |
|  |  | metabolism | 1 | 38 | 6.89 | 0.012 |  | -0.13 | -2.39 | 2.13 |
|  |  | metabolism*depletion type | 1 | 38 | 7.78 | 0.008 | CD | 4.47 | 1.23 | 7.71 |
| Lassitude | Ventral striatum, right | depletion type | 1 | 38 | 24.79 | <.0001 | CD | 2.69 | 1.60 | 3.78 |
|  |  | metabolism | 1 | 38 | 1.42 | 0.241 |  | -0.07 | -5.23 | 5.09 |
|  |  | metabolism*depletion type | 1 | 38 | 1.35 | 0.253 | CD | -5.04 | -13.82 | 3.75 |
|  |  |  |  |  |  |  |  |  |  |  |
| **Item** | **ROI** | **Effect** | **DF1** | **DF2** | **F-val** | **P-val** | **DT** | **Estimate** | **Lower** | **Upper** |
| Heart pounding | Pregenueal PFC, right | depletion type | 1 | 37 | 5.58 | 0.024 | CD | 0.55 | 0.08 | 1.03 |
|  |  | metabolism | 1 | 37 | 4 | 0.053 |  | 0.28 | -6.80 | 7.37 |
|  |  | metabolism*depletion type | 1 | 37 | 4.44 | 0.042 | CD | -11.18 | -21.93 | -0.43 |
| Hands trembling | PCC, left | depletion type | 1 | 37 | 7.81 | 0.008 | CD | 0.22 | 0.06 | 0.39 |
|  |  | metabolism | 1 | 37 | 22.81 | <.0001 |  | 0.00 | -1.50 | 1.50 |
|  |  | metabolism*depletion type | 1 | 37 | 22.81 | <.0001 | CD | 5.67 | 3.26 | 8.07 |
| work and activities | PCC, right | depletion type | 1 | 38 | 46.41 | <.0001 | CD | 1.63 | 1.15 | 2.12 |
|  |  | metabolism | 1 | 38 | 4.35 | 0.044 |  | 0.02 | -4.36 | 4.39 |
|  |  | metabolism*depletion type | 1 | 38 | 4.32 | 0.045 | CD | 9.22 | 0.24 | 18.19 |
| Feeling of choking | Anteromedial PFC, left | depletion type | 1 | 37 | 2.41 | 0.129 | CD | 0.10 | -0.03 | 0.23 |
|  |  | metabolism | 1 | 37 | 4.34 | 0.044 |  | 0.00 | -2.50 | 2.50 |
|  |  | metabolism*depletion type | 1 | 37 | 4.34 | 0.044 | CD | 3.98 | 0.11 | 7.85 |
| Depressed mood | Anterior insula, left | depletion type | 1 | 38 | 0.63 | 0.431 | CD | 0.16 | -0.25 | 0.57 |
|  |  | metabolism | 1 | 38 | 4.33 | 0.044 |  | -0.18 | -3.90 | 3.54 |
|  |  | metabolism*depletion type | 1 | 38 | 4.8 | 0.035 | CD | 7.23 | 0.55 | 13.91 |

# Table S2

|  | **Apparent sadness** | **Reported sadness** | **Concentration difficulties** | **Lassitude** | **Depressed mood** | **Work and activities** | **Feeling hot** | **Heart pounding** | **Feeling of choking** | **Hands trembling** |
| --- | --- | --- | --- | --- | --- | --- | --- | --- | --- | --- |
| DLPFC, left | **2.36** | 1.78 | 0.95 | -1.11 | **3.40** | -0.36 | -0.49 | 0.16 | 0.79 | -0.18 |
| DLPFC, right |
| Anterior PFC, left | **2.74** | **2.61** | 1.73 | 0.66 | 1.15 | 1.51 | **-3.01** | -0.58 | -0.78 | 0.30 |
| Anterior PFC, right |
| Hippocampus, left | -1.84 | -1.71 | **-2.20** | -0.09 | **-2.00** | -0.57 | **2.02** | 0.14 | -0.23 | -0.32 |
| Hippocampus, right |
| Ventral striatum, left | 1.27 | 1.45 | 0.56 | -1.15 | 0.89 | 0.19 | -0.16 | 0.64 | -0.22 | 0.57 |
| Ventral striatum, right |
| Pregenual PFC, left | 0.07 | 0.38 | -0.78 | -1.54 | 0.51 | -0.21 | 1.04 | 1.14 | 1.06 | 0.08 |
| Pregenual PFC, right |
| PCC, left | -1.95 | -1.90 | -1.83 | -1.23 | -0.94 | -1.46 | 1.71 | 1.64 | 0.79 | 1.26 |
| PCC, right |
| Anteromedial PFC, left | 0.49 | 0.06 | 0.05 | 0.23 | 0.16 | -0.56 | -1.55 | **2.42** | **2.18** | **2.05** |
| Anteromedial PFC, right |
| Anterior insula, left | 0.89 | 0.69 | 0.07 | 0.43 | 1.16 | 0.61 | -0.44 | 0.79 | 1.25 | 0.66 |
| Anterior insula, right |
